# Trade-off between local replication and long-distance dissemination during experimental evolution of a satellite RNA

**DOI:** 10.3389/fmicb.2023.1139447

**Published:** 2023-08-04

**Authors:** Shu-Chuan Lee, Ming-Ru Liou, Yau-Heiu Hsu, Ing-Nang Wang, Na-Sheng Lin

**Affiliations:** ^1^Institute of Plant and Microbial Biology, Academia Sinica, Taipei, Taiwan; ^2^Graduate Institute of Biotechnology, National Chung Hsing University, Taichung, Taiwan; ^3^Department of Biological Sciences, University at Albany, Albany, NY, United States

**Keywords:** bamboo mosaic virus (BaMV), satellite RNA (satRNA), satBaMV, experimental evolution, serial passage, antagonistic pleiotropy

## Abstract

Satellite RNAs (satRNAs) are molecular parasites that depend on their non-homologous helper viruses (HVs) for essential biological functions. While there are multiple molecular and phylogenetic studies on satRNAs, there is no experimental evolution study on how satRNAs may evolve in common infection conditions. In this study, we serially passaged the Bamboo mosaic virus (BaMV) associated-satRNA (satBaMV) under conditions in which satBaMV either coinfects an uninfected host plant, *Nicotiana benthamiana*, with BaMV or superinfects a transgenic *N. benthamiana* expressing the full-length BaMV genome. Single-nucleotide polymorphisms (SNPs) of satBaMV populations were analyzed by deep sequencing. Forty-eight SNPs were identified across four different experimental treatments. Most SNPs are treatment-specific, and some are also ephemeral. However, mutations at positions 30, 34, 63, and 82, all located at the 5′ untranslated region (UTR), are universal in all treatments. These universal SNPs are configured into several haplotypes and follow different population dynamics. We constructed isogenic satBaMV strains only differing at positions 30 and 82 and conducted competition experiments in protoplasts and host plants. We found that the haplotype that reached high frequency in protoplasts and inoculation leaves also exhibited poor dissemination to systemic leaves and vice versa, thus suggesting an apparent trade-off between local replication and long-distance dissemination. We posit that the trade-off is likely caused by antagonistic pleiotropy at the 5′ UTR. Our findings revealed a hitherto under-explored connection between satRNA genome replication and movement within a host plant. The significance of such a connection during satRNA evolution warrants a more thorough investigation.

## Introduction

Satellite RNAs (satRNAs) are a group of subviral agents that were first discovered by the presence of an extra, slower-sedimenting band during the ultracentrifugation step for the purification of the tobacco ringspot virus (TRSV) (Diener and Schneider, 1966; Schneider and Diener, 1966; Schneider, 1969; Annamalai et al., 2003). However, unlike the previously identified satellite tobacco necrosis virus (STNV) (Bawden and Pirie, 1942; Kassanis, 1962), the TRSV-associated satellite RNA (satTRSV) does not encode its own coat protein, a feature that differentiates between satellite viruses (SVs) and satRNAs. Regardless of the nomenclature, both SVs and satRNAs show little or no sequence homology to their cognate helper viruses (HVs) and rely entirely on HVs for fundamental functions, such as genome replication, encapsidation, and movement (Murant and Mayo, 1982; Francki, 1985; Roossinck et al., 1992; Simon et al., 2004; Hu et al., 2009; Palukaitis, 2016). As such, they are often considered parasites of viruses.

Between these two main groups of subviral agents, satRNAs, in particular, are exclusively associated with plant viruses. The effects of satRNAs on the infection cycles of their cognate HVs are multiple, including attenuation of symptoms caused by HVs (Li and Simon, 1990; Collmer and Howell, 1992; Taliansky and Robinson, 1997; Hsu et al., 1998), alteration of HV RNA accumulation (Gal-On et al., 1995; Hsu et al., 1998), enhancement in HV movement (Scholthof, 1999; Zhang and Simon, 2003) or transmission (Naidu et al., 1999; Robinson et al., 1999; Anitha et al., 2014; Jayasinghe et al., 2021). Numerous satRNA isolates obtained in the fields also provided valuable information on satRNA biology. For example, comparative analyses and experimental validations have been used to identify sequences and structures important for satRNA replication (Carpenter and Simon, 1998; Annamalai et al., 2003; Guo et al., 2009; Chen et al., 2012; Murawski et al., 2015) and to demonstrate the trade-offs between satRNA transmission and virulence in an epidemic (Escriu et al., 2000a,b). In addition, phylogenetic analyses of field samples have traced the evolutionary dynamics of satRNA isolates during a disease outbreak (Grieco et al., 1997; Garcia-Arenal et al., 2000) and characterized the population genetic structure and geographic distributions of various satRNAs (Liu et al., 1997; Pinel et al., 2003; Wang et al., 2014, 2017). Evolutionary studies, in the form of serial passage experiments, provided a possible mechanism for the generation of sequence diversity observed in the field (Kurath and Palukaitis, 1989, 1990) and potential pathways for the *de novo* evolution of satRNA sequences (Hajimorad et al., 2009). However, as far as we know, there is no study on how satRNAs would evolve when encountering different infection ecology, e.g., coinfection of an uninfected host with the HV or superinfection of an HV-infected host, as discussed in the case of the hepatitis D virus (a satellite) and hepatitis B virus (the helper) (Negro, 2014).

SatRNA of *Bamboo mosaic potexvirus* (satBaMV) was initially isolated from a BaMV-infected bamboo (Lin and Hsu, 1994). BaMV has a positive single-stranded RNA genome of approximately 6.4 kilobases [excluding the poly(A) tail], which consists of 94-nucleotide (nt) 5′ untranslated region (UTR), 142-nt 3′ UTR, and five open reading frames (ORFs). The BaMV ORF1 encodes viral replicase, ORF2-4 are three overlapped genes that encode viral movement proteins (MPs), called triple gene block protein 1 (TGBp1), TGBp2, and TGBp3, and ORF5 encodes the coat protein (CP) for virion formation, viral movement, and symptom formation (Lin et al., 1994; Lan et al., 2010). The satBaMV genome is also a positive single-stranded RNA of 836 nts (excluding the poly(A) tail) in length that is composed of 159-nt 5′ UTR, 125-nt 3′ UTR, and 552-nt ORF which encodes a 20-kDa nonstructural protein, P20 (Lin and Hsu, 1994). The P20 protein is not required for satBaMV replication (Lin et al., 1996); still, it preferentially binds to satBaMV RNA (Tsai et al., 1999), which facilitates the systemic movement of satBaMV RNA in the host plant through interaction with a host nucleolar factor, fibrillarin (Palani et al., 2006; Vijayapalani et al., 2012; Chang et al., 2016). Several field isolates showed a remarkable interfering phenotype, manifested as reduced BaMV RNA accumulation and attenuated BaMV-induced symptoms (Hsu et al., 1998, 2006; Annamalai et al., 2003; Chen et al., 2007). The key determinant of the interference was mapped to the 5′ UTR in a conserved and functionally interchangeable apical hairpin stem-loop (AHSL) (Yeh et al., 2004; Chen et al., 2007). Further studies showed that a single nucleotide substitution from U to C at position 82 in the AHSL region was sufficient to result in the phenotypic switch from non-interfering to interfering, with an accompanying replication advantage over other satBaMVs (Chen et al., 2007, 2012). Since the reduction of BaMV replication by interfering satBaMV is dose-dependent, it implies that there is competition for limited replication components/complexes between BaMV and satBaMV (Chen et al., 2007; Chou et al., 2013). Likely, the structural similarities between satBaMV and BaMV at the conserved AHSL in the 5′ UTR and three stem-loops at the 3′ UTR contribute to the recruitment of replication components/complexes, albeit no sequence similarity is shared between these entities (Annamalai et al., 2003; Yeh et al., 2004; Chen et al., 2007, 2010; Huang et al., 2009).

In the field, BaMV can be transmitted through the vegetative growth of bamboo rhizomes or mechanical injury during the harvesting of bamboo shoots or feeding by insect vectors (Chang et al., 2017). Since satBaMV is only found in BaMV-infected bamboos (Liu et al., 1997; Lin et al., 2015), its transmission will either result in coinfection, with BaMV, of uninfected plants or superinfection of plants already infected with BaMV. Although many satBaMV isolates are analyzed, how satBaMVs adapt to different infection conditions is still largely unknown.

In this study, to mimic the conditions satBaMVs are likely to encounter in the field, we set up an experimental system to investigate the evolution of satBaMV by serial passage under two infection conditions: coinfection of uninfected hosts with its HV BaMV and superinfection of hosts already with a resident BaMV present. The evolutionary dynamics were analyzed using deep sequencing of the satBaMV and BaMV populations. Significant nucleotide substitutions were identified, and their roles in satBaMV evolution were assessed through *in vivo* replication and movement assays. Our results showed that experimental evolution with serial passages recapitulates known nucleotide substitutions that are important for satBaMV replication. They also revealed an unexpected trade-off between replication and movement via antagonistic pleiotropy.

## Materials and methods

### Plant materials and growth conditions

All plants were grown in a walk-in growth chamber at 28°C with a 16-h light/8-h dark cycle. Three- to four-week-old *N. benthamiana* and five-week-old *C. quinoa* plants were used for BaMV and satBaMV inoculation.

The generation of transgenic *N. benthamiana* was performed as previously described (Chou et al., 2013). Briefly, *Agrobacterium tumefaciens* C58C1 was used to introduce the BaMV infectious cDNA clone, pKB (Liou et al., 2014) (Supplementary Figure S1), into *N. benthamiana*. BaMV-positive transgenic lines were screened by detecting the expression of the viral coat protein (CP) gene. A homozygous second filial generation (F_2_) of the symptomatic line 27–17 was used in this study (see Supplementary Figure S1 for other lines not chosen).

### Experimental evolution and sample collection

**Figure 1:**
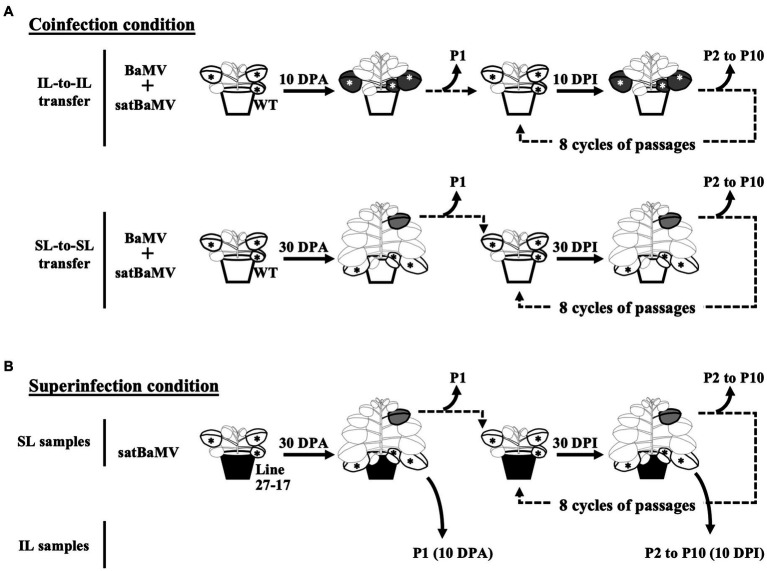
Experimental design for serial passage experiments. *N. benthamiana* plants were first infected by agroinfiltration. The subsequent passages were conducted by mechanical inoculation of tissue sap rubbed onto new plants. Sampling and passaging were performed in the inoculation leaf (IL) at 10 days interval and the sixth systemic leaves (SL) at 30 days interval. Ten passages were conducted at the indicated time points (DPA, days post agroinfiltration; DPI, days post sap inoculation). Passages for **(A)** coinfection and **(B)** superinfection conditions are shown. The satBaMV populations were analyzed through Illumina deep-sequencing. Star symbols indicate the ILs of agroinfiltration or sap inoculation; green leaves the sampled ILs; grey leaves the sampled SLs.

Experimental designs for the serial passage experiments are shown in Figure 1. All plants used are three to four weeks old. The initial inoculation was conducted by agroinfiltration with *A. tumefaciens* C58C1 containing pKB or pKF4 (Liou et al., 2014) on four *N. benthamiana* plants. Inoculated leaves (ILs) of four inoculated plants were collected and pooled together at 10 days post-infection (DPI) and designated as IL passage 1 (IL-P1). The systemic leaves (SLs, the sixth leaf above IL) were collected and pooled at 30 DPI and designated SL-P1. The pooled leaves were cut into small pieces for further processing. Half of each P1 sample was frozen with liquid nitrogen and stored at −80°C for satBaMV population analysis. The other half was immediately ground with 10 volumes of sodium phosphate buffer (10 mM, pH 7.0) with a pre-chilled mortar and pestle. The resulting crude sap was then used to inoculate four *N. benthamiana* plants for the next round of infection with mechanical means. Leaf samples were collected at 10 DPI for IL and 30 DPI for SL, as described above, and designated P2. A total of 10 passage (P1–P10) samples were collected.

### RNA extraction and detection

Total RNAs were extracted from 0.1 g frozen leaf samples using TriPure Isolation Reagent, following the manufacturer’s instructions (Roche Diagnostics GmbH, Germany). RNA quality and quantity were determined by NanoDrop ND-1000 Spectrophotometer (Thermo Fisher Scientific Inc., United States). RNA blot was performed following the instruction of DIG DNA Labeling and Detection Kit (Roche, Germany). Briefly, 1 μg of total RNA was separated on a 1% SeaKem® LE agarose gel (Lonza Rockland, Inc., United States) in 0.5X TBE buffer (OmicsBio., Taiwan). RNA was then transferred onto a Hybond-N+ nylon membrane (GE Healthcare Life Sciences, United Kingdom). Transferred blots were hybridized with DIG-labelled probes against BaMV CP-3′ UTR or BSF4 genomes (Hsu et al., 2000). BaMV or satBaMV was detected with AP-conjugated anti-DIG antibody and CDP-star (Roche Diagnostics GmbH, Germany). The signal was recorded through X-ray film exposure. Images were edited by Adobe Photoshop CS6.

### SatBaMV cDNA library construction and Illumina deep sequencing

The satBaMV amplicon was obtained through RT-PCR. Briefly, 1 μg of plant total RNA from passaged samples was used for the first-strand cDNA synthesis using SuperScript III reverse transcriptase (Invitrogen, United States) and primer A02-R (targeting the 3′ end of the satBaMV genome). For the control sample (Tx), total RNA from healthy plants was spiked with 100 ng BSF4 *in vitro* transcripts. The resulting first-strand cDNAs were then used as templates for generating full-length satBaMV amplicons by PCR using primers A02-R and A01-F (targeting the 5′ end of the satBaMV genome) (Lin and Hsu, 1994). PCR reaction was performed by Phusion Flash High-Fidelity PCR Master Mix (Thermo Fisher Scientific, Inc., United States), following the manufacturer’s instructions. The satBaMV amplicon was purified by QIAEX II Gel Extraction Kit (QIAGEN, United States) and used for pair-end (2 × 300 bp) libraries preparation using standard Illumina protocols with recommended barcode sets. The libraries were sequenced using Illumina MiSeq System (Illumina, United States) in High Throughput Genomics Core, Biodiversity Research Center, Academia Sinica, Taiwan.

While all relevant samples in each passage were collected, limited samples were subjected to deep sequencing due to limited resources. The analyzed samples are (1) P1, P9, and P10 for the Co-SL passage, (2) P1, P9, and P10 for the Su-SL passage, (3) P1, P2, P6, and P10 for the Co-IL passage, and (4) P1, P2, P6, and P10 for the Su-IL passage. The prefix “Co” denotes the viral populations derived from coinfection of both BaMV and satBaMV, while the “Su” populations were obtained from superinfection of the transgenic line 27–17 plants.

### Bioinformatics analysis

The Illumina raw reads were preprocessed using Trimmomatic 0.35 (Bolger et al., 2014) to remove Illumina adaptor sequences and quality trimming (with setting SLIDINGWINDOW:5:20 MINLEN:150), where only reads longer than or equal to 150 bps were kept. Removal of customized adaptor sequences, the flanking, and oligo-dT regions in the primer ends used for amplicon generation was performed using cutadapt 1.9.1 (Martin, 2011). Preprocessed reads were mapped to the satBaMV sequence (Lin and Hsu, 1994) using BLAT (Kent, 2002) with default parameters and alignments filtered by a threshold identity of 90%. SNV/InDel calling was performed using RackJ.^1^ SNVs were counted based on the qualified reads that mapped to the satBaMV genome continuously, while InDels were counted based on qualified reads that mapped to the satBaMV genome discontinuously. For a more extensive analysis of satBaMV’s 5′-end, only reads covering nucleotide positions 30 to 82 were selected. The reads were clustered into all possible combinations of haplotypes in nucleotide positions 30, 34, 63, and 82, based on the BSF4 genomic sequence (Lin and Hsu, 1994).

### Generating satBaMV mutations in the 5′ UTR

Plasmid pCF4 is an infectious cDNA clone of satBaMV BSF4, the expression of which is driven by the 35S promoter (Lin et al., 2004). QuickChange II Site-Directed Mutagenesis Kit was used to introduce selected mutations in the 5′ UTR to plasmid pCF4. Identities of the introduced mutations were confirmed by sequencing. The naming of these mutant plasmids is based on the nucleotide positions. For example, pCF4.30A82C denotes that nucleotide positions 30 and 82 are A and C, respectively. A total of four mutant plasmids were constructed: pCF4.82C, pCF4.30C82C, pCF4.30A82C, and pCF4.30A82A. These mutants were also subcloned from pCF4 into a binary vector pKn, as described for pKF4 (Liou et al., 2014), for agroinfiltration in plants and named pKF4.82C, pKF4.30C82C, pKF4.30A82C, and pKF4.30A82A, accordingly.

### *In vivo* competition experiment with protoplast

Protoplasts were isolated from 30-day-old *N. benthamiana* leaves (Chen et al., 2012). A total of 10 μg plasmids with an equal amount of pCF4 and the four mutant plasmids described above were mixed with 5 μg of pBaORF1 and co-inoculated into 4 × 10^5^ protoplasts. The plasmid pBaORF1 was used to provide replicase for satBaMV replication (Prasanth et al., 2011; Chen et al., 2012). Inoculated protoplasts were incubated at 25°C with continuous light for 2 days. Harvested protoplasts were used for total RNA extraction and RNA blot analysis of satBaMV accumulation as described above. After the competition assay, the populations of satBaMV progeny were analyzed by cloning and sequencing. The satBaMV amplicons were PCR-amplified, as described above, and cloned into the pJET1.2/blunt cloning vector (CloneJET PCR Cloning Kit, Thermo Fisher Scientific, Inc. United States). Twenty to thirty individual clones in each competition assay were used for sequencing to enumerate the haplotype frequency.

### *In planta* competition experiment

For *in planta* competition, agroinfiltration of BaMV and satBaMV were performed. *Agrobacterium* C58C1 containing pKB, pKF4, or four pKF4 variants was cultured separately, and the bacterial suspensions were adjusted to OD_600_ = 1 for use. Agroinfiltration was performed by mixing an equal amount of bacterial suspension containing pKB with pKF4 or a mixture of an equal ratio of pKF4 and four variants to a final concentration of OD_600_ = 1. Leaf tissues collected at 10 DPI for IL and 30 DPI for SL were used for total RNA extraction and satBaMV progeny analysis. Again, the haplotype frequencies were enumerated by cloning and sequencing, as described above.

### Statistical analyses

RStudio ver. 1.3.959 was used for all statistical analyses. Specifically, the *fisher.test* function in the R Stats Package *stats* ver. 3.3.3 was used for Fisher’s exact test of independence. The *xmulti* and the *binom.test* functions, both in the *XNominal* package ver. 1.0.4, were used for the exact test of independence and *post hoc* analyses, respectively.

## Results

### Effects of coinfection and superinfection on satBaMV replication

**Figure 2:**
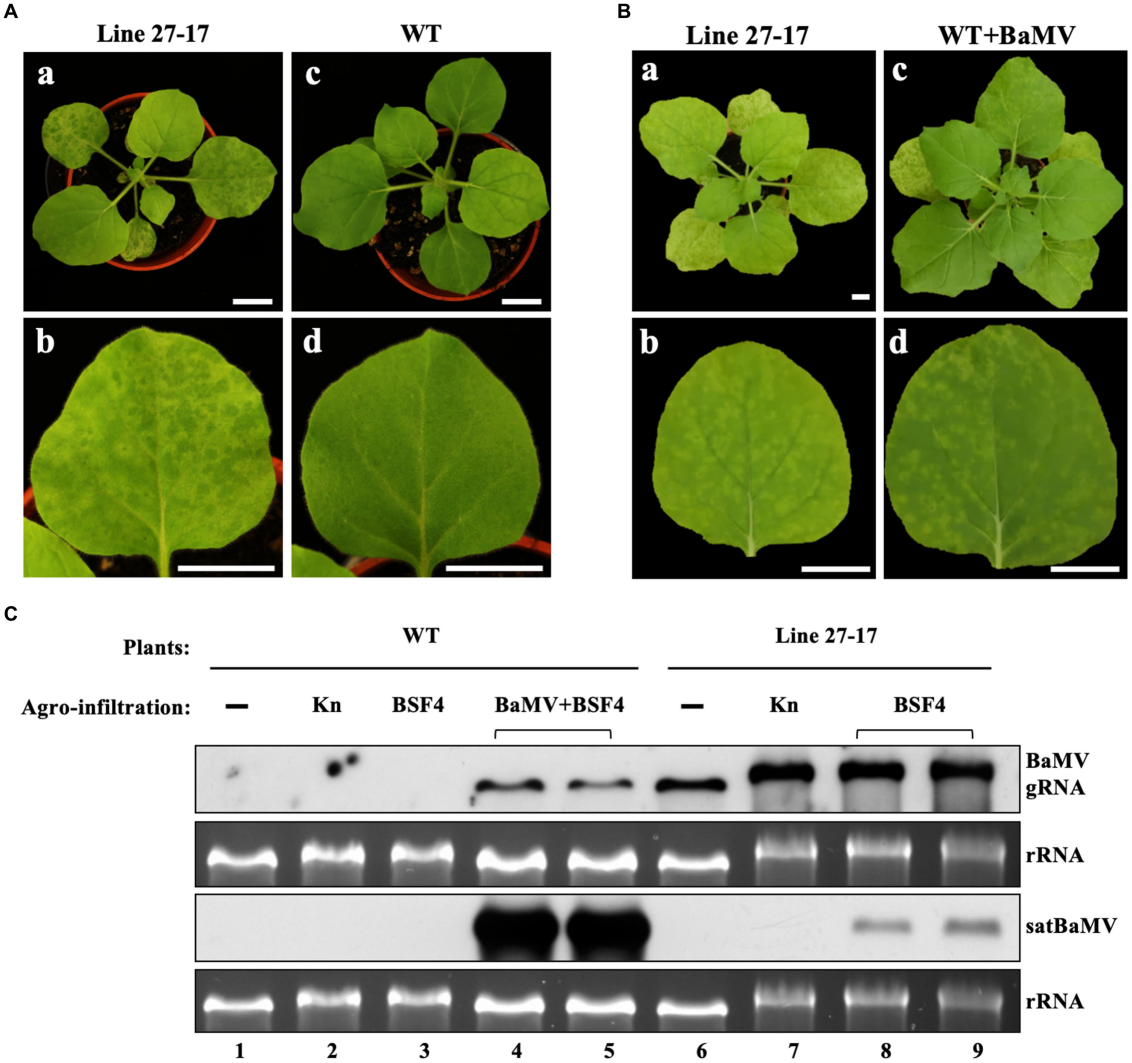
Accumulation of BSF4 satBaMV in BaMV-transgenic (Line 27–17) and wild type (WT) *N. benthamiana* plants. **(A)** Phenotypes in whole plants (a and c) and fully expanded leaves (b and d) of 22-day-old Line 27–17 (a and b) and WT (c and d) plants. **(B)** The similar symptoms of Line 27–17 and BaMV-infected WT plants. The 22-day-old WT plants were agroinfiltrated with *Agrobacterium* carrying pKB. BaMV symptoms at 14 days post agroinfiltration (DPA) are shown. The photographs were taken for whole plants (a and c) and systemic leaves (b and d), respectively. **(C)** Accumulation of BaMV and BSF4 in WT and transgenic line 27–17 plants. The WT and transgenic plants were agroinfiltrated, as described previously. The BaMV and satBaMV accumulated in the inoculation leaves (IL) were assayed at 10 DPA by RNA blots with DIG-labeled probes against the BaMV CP gene or satBaMV genome, respectively. “▬”, non-infiltrated mock plants; Kn, *Agrobacterium* containing expression vector pKn as control; BSF4, *Agrobacterium* containing satBaMV infectious cDNA clone, pKF4; BaMV, *Agrobacterium* containing BaMV infectious cDNA clone, pKB. Horizontal bracket symbols indicate two independent results. Different treatments were also indicated by the labeled number 1–9 below the RNA panels. Bars in **(A)** and **(B)** equal 2 cm.

To explore the impact of coinfection and superinfection on the replication of the satellite RNA, we used BaMV (Bamboo mosaic virus) and its associated satellite RNA (satBaMV) as the model system and *Nicotiana benthamiana* as the host plant. To mimic the constant presence of a resident HV, we generated transgenic lines of *N. benthamiana* that constitutively express the full-length BaMV genome (Supplementary Figure S1). As shown in Figures 2A,B, the transgenic line 27–17 shows a similar leaf symptom to the BaMV-infected wild-type plant, demonstrating that line 27–17 sustains an active infection of BaMV (Figure 2C, lane 6).

To realize the coinfection scenario, we used the agroinfiltration method to introduce plasmids expressing satBaMV and BaMV into uninfected wild-type host plants. To simulate the superinfection scenario, the plasmid expressing satBaMV was agroinfiltrated to the transgenic line 27–17. As shown in Figure 2C, when compared to the coinfection condition, the satBaMV BSF4 isolate showed reduced replication under the superinfection condition (rRNA-standardized satBaMV transcripts reduced to only 13% and 25%), with a concomitant increase of the helper BaMV (rRNA-standardized BaMV transcripts increased 3.9 and 5.7 folds). This result suggests that BSF4 isolate is likely subjected to more severe competition for replication under the superinfection condition.

### Serial passage experiments and expectations

Prompted by the observation that satBaMV replicates differentially under coinfection and superinfection conditions, we decided to investigate how satBaMV would evolve under these two infection scenarios we envisioned. Serial passage experiment (SPE), a common practice for studying evolution in a laboratory setting, was adopted for this purpose. Besides evolution in different infection conditions, we also expanded our study to include the contrast between evolution under the condition of local replication vs. long-distance dissemination. The rationale is that during either vector or mechanical transmission of plant viruses, the source of the transmitted viruses is often from systemic leaves that are some distance away from the initial inoculation site to which the original viral population is introduced.

Figure 1 shows the experimental setup for these SPEs. Operationally, both coinfection (“Co”) and superinfection (“Su”) were initiated by agroinfiltration with plasmids expressing full-length genomes of satBaMV and/or BaMV. Subsequent infections were performed by rubbing the sap of diseased leaves onto the carborundum-dusted leaves of the new plants. The passage interval for the inoculation leaf (“IL”) passage is 10 days after infection and 30 days for the systemic leaf (“SL”) passage. A total of 10 passages (P1–P10) were conducted for each SPE. It is important to note that the Co-IL and Co-SL populations are from two independent SPEs, while the Su-IL and Su-SL populations are in the same temporal sequence.

We hypothesized that the selective pressure on satBaMV could be quite different between Co and Su or between local replication (using “IL” as a proxy) and long-distance dissemination (using “SL” as a proxy). Since the accumulation of the satBaMV is reduced more under the Su than the Co condition (Figure 2C), this observation suggests that satBaMV experiences more severe competition for replication during superinfection. Furthermore, it is known that the 5′ UTR (nts 1–159) and the satBaMV-encoded P20 (nts 160–708) protein contribute to the systemic trafficking of satBaMV (Palani et al., 2006). Therefore, if the main differentiating factor for the Co and Su scenarios is the level of replication stress, then we would expect to observe evolution at the 5′ UTR and 3′ UTR as a response to such stress, especially at the AHSL (nts 54–91), which is critical for satBaMV genome replication (Annamalai et al., 2003; Yeh et al., 2004; Hsu et al., 2006; Chen et al., 2012). Similarly, we also expect to observe SPE-specific mutation distributions at the *P20* gene when comparing local replication and long-distance dissemination scenarios.

### Identification of single-nucleotide polymorphism

Leaf samples were collected during the passage experiments. The satBaMV populations were extracted, reverse-transcribed, amplified with PCR, then deep-sequenced.

Analyses of the deep-sequencing results showed that more than 99% of the reads are unambiguous and can be mapped to the BSF4 genome, with an average read-depth of 376,251 per site (median: 375,221; range: 275,093–413,536) (Supplementary Table S1).

To establish the baseline of sequencing noise resulting from nucleotide misincorporations during reverse transcription, PCR amplification, and deep sequencing, we included a spiked control sample with RNA transcripts derived from a cDNA clone of the ancestral satBaMV strain, BSF4 (GenBank accession number: AY205227). For example, in our control sample, the read-depth at position 82 is 515,395; 174 showed the U82A, 1,199 the U82C, and 851 the U82G reads, with frequencies of 0.0003, 0.0023, and 0.0017, respectively. The combined misincorporation rate at this particular position is therefore summed up to be 0.0043. On average, for the satBaMV genome, the combined misincorporation rate per nucleotide position is 0.0026 ± 0.0017 (std. dev.) with a range of 0.0004–0.0214. Interestingly, the observed misincorporation rates at each nucleotide position vary greatly along the entire satBaMV genome, with the elevated rates clustered at the 5′ and 3′ UTRs (see Supplementary Figure S2).

To identify sequence variations that are likely due to evolutionary processes rather than nucleotide misincorporations during sample preparations, we applied an arbitrary but conservative criterion by defining a specific base-calling a single-nucleotide polymorphism (SNP) if the base-calling rate of any particular variant—after discounting the corresponding rate in the control sample—is ≥0.01. For example, for the Co-IL-P1 sample, the uncorrected frequency for U82C mutation is 0.0677, while the corresponding change in the control sample is 0.0023. The corrected frequency for the U82C mutation is then calculated as 0.0654 for this specific sample (satBaMV population). The exceptions are positions 697, 749, 768, 807, 817, and 828, all of which have misincorporation rates ≥0.01 and thus are excluded from the analyses.

Based on the criterion established above, we identified 48 SNPs among 45 sites that have net frequencies (i.e., the base-calling rates after subtracting from the corresponding rates from the control sample) that are ≥0.01 (see Supplementary Table S2).

### Patterns of SNP distributions

**Figure 3:**
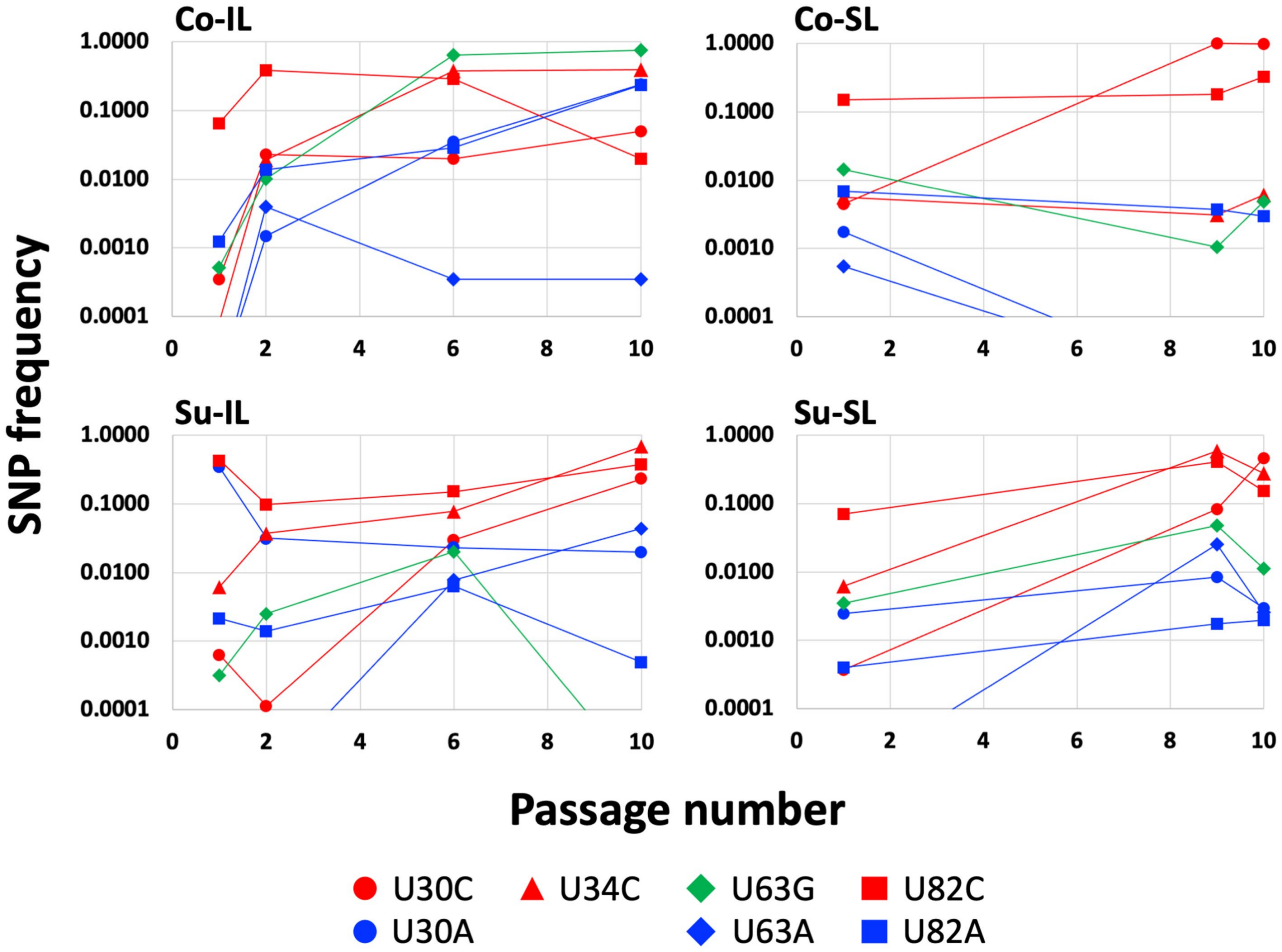
Changes of SNP frequencies in the satBaMV 5′ UTR. SNPs at four notable polymorphic sites (positions 30, 34, 63, and 82) in the 5′ UTR were plotted for the Co-IL, Co-SL, Su-IL, and Su-SL SPEs. Only frequencies ≥0.01 are identified as SNPs. This figure is a graphical representation of part of the data in the Supplementary Table S2. Frequencies lower than 0.01 were plotted for graphing purpose.

A closer inspection of the SNP distribution among the experimental sets of Co-IL, Co-SL, and Su-IL/Su-SL (see Supplementary Table S2) revealed several interesting patterns. Of the eight SNPs in the 5′ UTR, three (U30C, U63G, and U82C) appeared in all satBaMV populations. Among these three SNPs, U82C is the only one that occurred early in P1 and persisted to P10. The U30C mutation appeared in later passages but persisted to P10 as well. The U63G has a similar pattern to U30C in the Co-IL and Su-SL populations, but not the others. There is no discernible pattern when comparing the Co and Su samples. However, it is notable that the U30A mutation only appeared in the IL samples. The appearances of the remaining four SNPs are idiosyncratic; some appeared only in one SPE (U82A in Co-IL) or just one passage (U63A in Su-SL-P9 and C148U in Co-IL-P6). It is interesting to note that the presence of U34C is almost universal and persistent, except for the Co-SL passages. Figure 3 shows the changes of notable 5′ UTR SNP frequencies during each passage experiment.

Of the 36 SNPs in the *P20* region, 28 only appeared in one of the satBaMV populations; none appeared in all. Furthermore, many SNPs are ephemeral and appear only once in the sampled time points, often in the intermediate passages and at relatively low frequencies. The exceptions are U215G (resulting in P20 I19S change) and U317C (resulting in P20 V53A change) in the Co-IL populations. These two SNPs showed increased frequencies from ∼1% in P6 to ∼20% in P10. Another exception is the U661C mutation (resulting in P20 Y168H change) of the Su-IL/Su-SL set. This SNP is maintained from P6 to P10 at ∼75–85%. Most SNPs in the 3′ UTR are ephemeral and relatively low in frequencies. The only exception is the G799A mutation in the Su-IL/Su-SL set, with its frequency increased from ∼5% to ∼25% within one passage.

**Figure 4:**
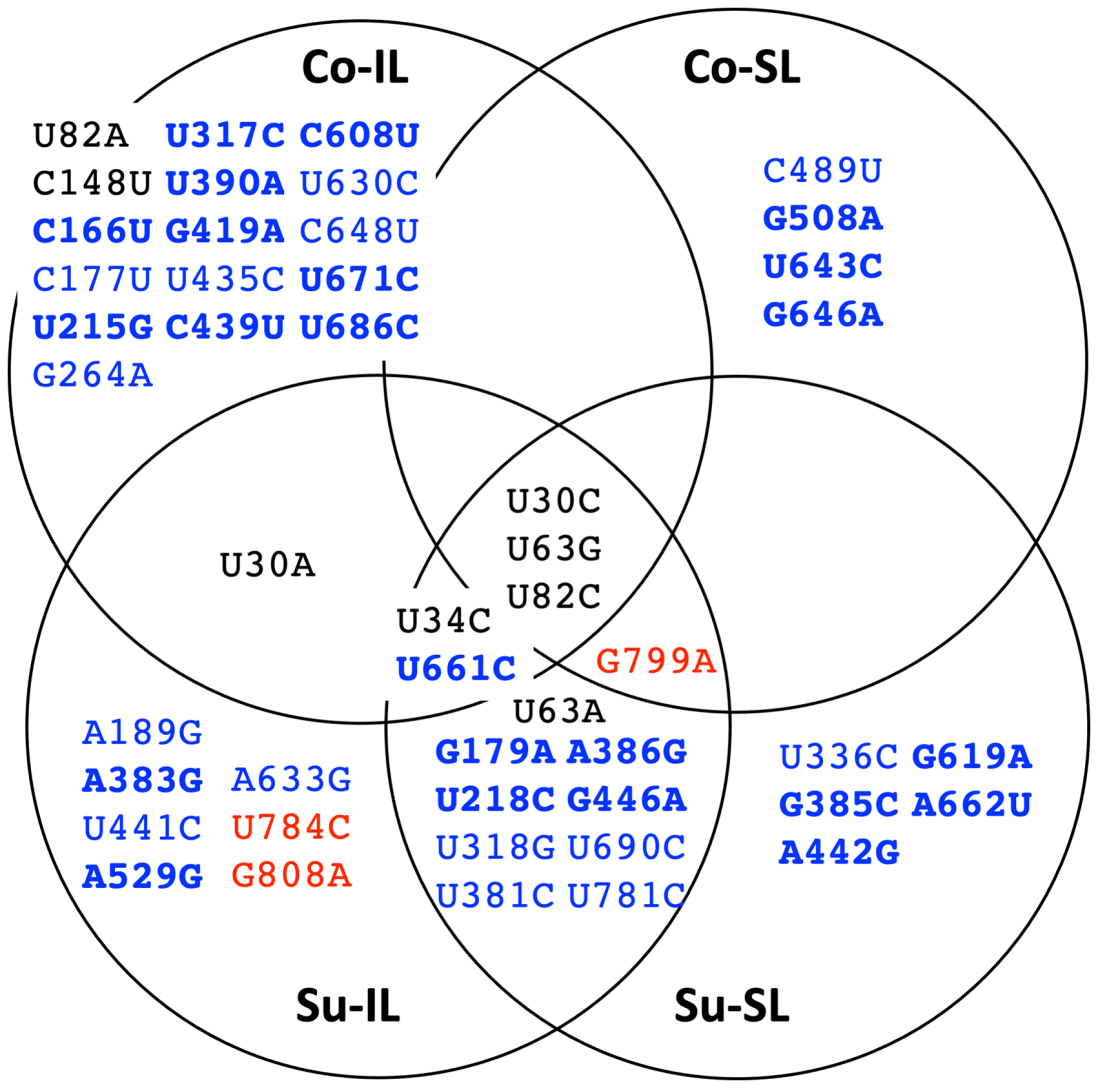
Summary distribution of satBaMV SNPs among serial passage experiments. Only mutant frequencies ≥0.01 are identified as single-nucleotide polymorphisms (SNPs). Each of the 48 SNPs is placed in the serial passage experiment from which it was found. Black font indicates SNPs at the 5′ UTR, blue the *P20* gene, and red the 3′ UTR. Boldface font indicates mutation resulting in amino acid replacement. See Supplementary Table S2 for details.

The distribution of all 48 SNPs among the SPEs is summarized as a Venn diagram, shown in Figure 4. Ostensibly, a great majority of the SNPs are unique to one of the SPEs (33 SNPs, ∽69%), suggesting the importance of these SNPs for satBaMV fitness is context-dependent. Also, among the remaining shared 15 SNPs, we do observe two interesting patterns: (1) there are eight SNPs, all in the *P20* gene, that are only found in the Su sample but not the Co samples, and (2) there is one SNP (U30A) that is only found in the IL samples, but not the SL samples.

### Haplotype frequencies and dynamics in the 5′ UTR

Since positions 30, 34, 63, and 82 in the 5′ UTR are close to each other and approximately a quarter (median: 28.0%; range: 24.97%–28.61%) of mapped sequencing reads covered all four sites, we were able to obtain haplotype frequencies in this specific region among all passage experiments without ambiguity. Only haplotypes that have once reached ≥0.01 in frequency are recorded (after correction for nucleotide misincorporation as described previously). The haplotype frequencies are shown in Supplementary Table S3.

**Figure 5:**
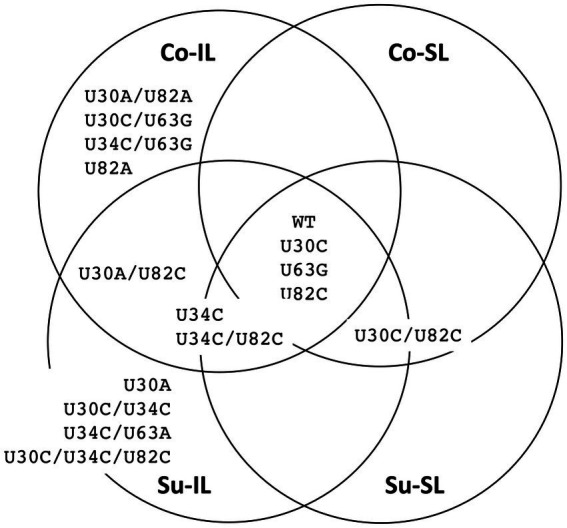
Summary distribution of satBaMV 5′ UTR haplotypes among serial passage experiments. Only haplotype frequencies ≥0.01 are shown. Each of the 15 mutant haplotypes is placed in the serial passage experiment from which it was found. See Supplementary Table S3 for details.

Of 256 possible permutations, 16 haplotypes (including the ancestral type) were identified among all SPEs passages (Figure 5). Each Co and Su experiment has 12 haplotypes; eight are shared. Almost twice as many haplotypes are found in the IL samples (11 and 12 for the Co and Su conditions, respectively) than in the SL samples (5 and 7, respectively). Furthermore, all haplotypes in the SL samples are a subset of those in the corresponding IL samples, except for the U30C/U82C haplotype, which appeared in the Co-SL sample but not Co-IL. The most parsimonious explanation is that most of the haplotypes in the SL are disseminated from the initial IL rather than mutations that appeared *de novo* in SL.

**Figure 6:**
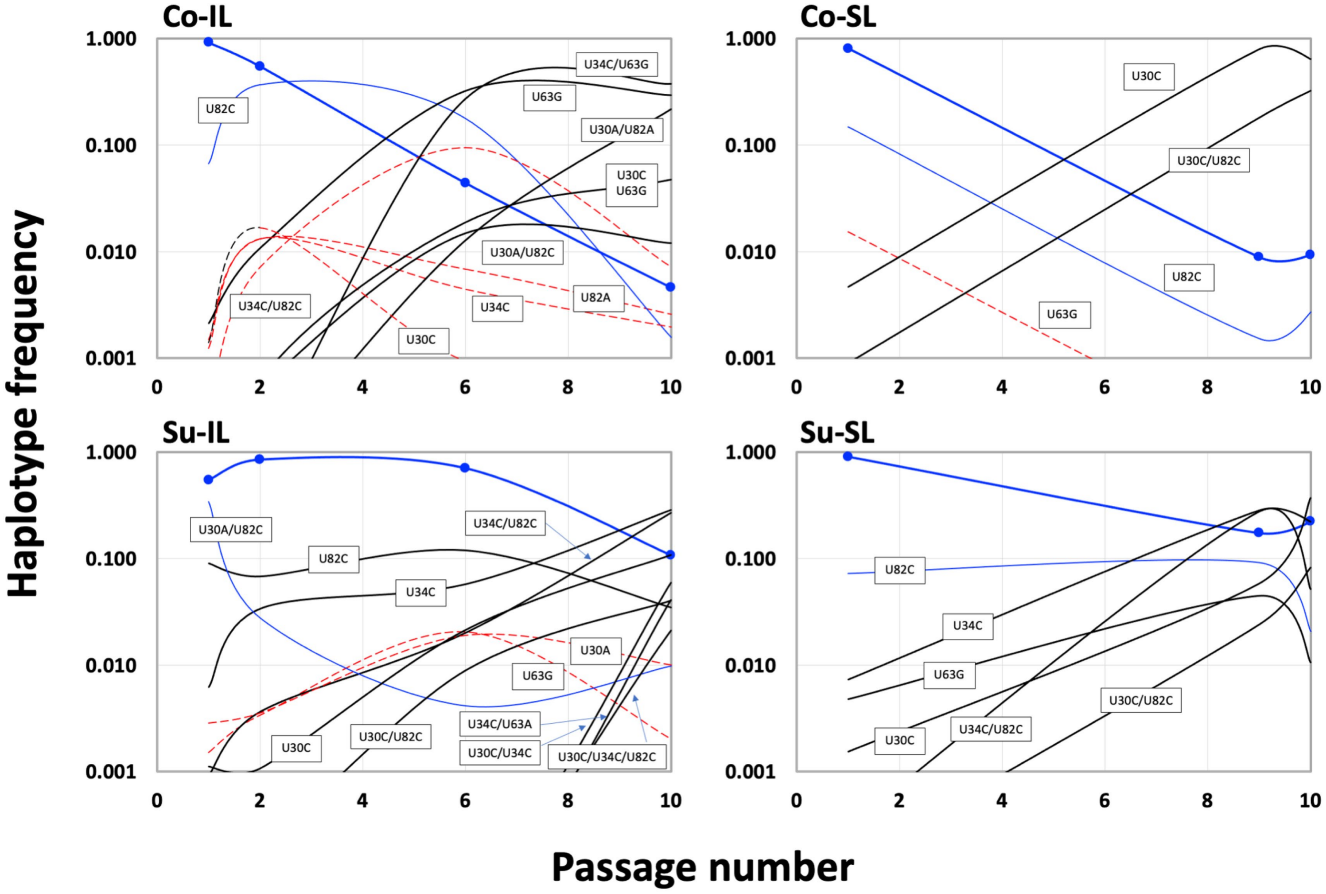
Population dynamics of satBaMV 5′ UTR haplotypes among serial passage experiments. Haplotype frequency is plotted against the passage number. Blue curves show the haplotypes with a decreasing trend during the passages, dashed red curves show the rise-and-fall pattern, and black curves show an increasing trend (see text for details). Thick blue curves show the frequencies of the ancestral BSF4, and blue symbols indicate the passage numbers sampled. Although Figure 3 only listed the haplotypes with frequencies ≥0.01, this figure shows the frequencies <0.01 to demonstrate the changes in haplotype frequencies.

Temporally, we can discern three groups of haplotypes in all SPEs (Figure 6). The early group (defined as those haplotypes with frequencies ≥0.01 at P1; blue lines in Figure 6) is composed of the ancestral BSF4 (U in all four positions), U82C, and its derivative, U30A/U82C. All early group members started with high frequencies in P1 and declined steadily during passaging. However, the extent of the decline is not the same. Under the Co condition, the frequencies of these early haplotypes dropped below 0.01 at P10. However, in the Su condition, all frequencies are higher than 0.01; one (the BSF4 in the Su-SL) is even maintained at ∽0.22 at P10.

The intermediate haplotype group showed a rise-and-fall pattern during the passages (red dashed lines in Figure 6). Interestingly, the maximum frequency for this group never exceeded 0.10. At P10, their presence is generally below our prescribed threshold of 0.01. Their decline is accompanied by the emergence of the third, late group that eventually reached high frequencies at P10 (black lines in Figure 6). None of the haplotypes in the late group that reached a considerable frequency, say ≥0.1, at P10 showed appreciable frequency at P1. The haplotypes in the early and intermediate groups tend to be one mutational step away from the ancestral sequence, while those in the late group are predominantly two steps away.

Since the U82C mutation is critical for satBaMV replication competence (Chen et al., 2012), the early emergence of the U82C haplotype in all P1 samples with high frequency shows that selection for more efficient replication is a crucial first step in satBaMV evolution, irrespective of experimental conditions. However, like the ancestral haplotype, the U82C haplotype also suffered a universal decline in all SPEs (see Supplementary Table S4). Nevertheless, the appearance of other mutations, such as U30C, on the U82C background allows the U82C to hitchhike to a higher frequency, thus maintaining its presence in P10 in all experimental conditions. From the result, it is also clear that non-U82C mutations can achieve high frequencies in P10, although the exact identities of these non-U82C mutations depend on the experimental conditions.

### Local replication and long-distance dissemination during SPEs

For plant viruses, an initial infection at a local tissue often leads to systemic infection of the entire plant. All else being equal, it is reasonable to assume that a genotype that maintains a higher frequency in the population at the initial infection site will have a higher chance of being disseminated to distant parts of the plant for secondary infections. That is, if the sole driving force for the evolution of satBaMV is simply its ability to maintain a higher frequency throughout the plant (thus ensuring its transmission to the next host plant), then we should expect the rankings of genotype frequencies to be concordant between the IL and SL sampling sites.

For the Co condition, the dominant haplotypes (with frequencies ≥0.1) in P10 are U34C/U63G (0.377), U63G (0.294), and U30A/U82A (0.217) for the Co-IL passage and U30C (0.640) and U30C/U82C (0.326) for the Co-SL passage (Figure 6 and Supplementary Table S3). These two sets of haplotypes are categorically different from each other. For the four shared haplotypes between the Co-IL and Co-SL throughout the passages, two of them (the ancestral BSF4 and U82C) showed a steady decline in both passages. The other two showed opposite trends; the U63G frequency increases in the IL passage but decreases in the SL passage, while the U30C frequency decreases in the IL passage but increases in the SL passage (Figure 6 and Supplementary Table S3). The contrast between Co-IL and Co-SL passages suggests that the U63G mutation appears advantageous in the IL passage but not the SL passage. More interestingly, the U-to-A mutation at positions 30 and 82 seemed beneficial for IL passaging. In contrast, the U-to-C mutation at the same two positions is advantageous for SL passaging.

For the Su condition, however, the result is different. While samples from the Co-IL and Co-SL passages are independent, the samples from the Su-IL and Su-SL are temporally linked (see Figure 1). The Su-IL-P1 sample precedes the Su-SL-P1 sample, which in turn precedes the Su-IL-P2 sample, which is then followed by the Su-SL-P2 sample. This relationship provides a more direct link between samples from the Su-IL and Su-SL. For the samples we investigated, we can compare the temporal sequences of Su-IL-P1 → Su-SL-P1 → Su-IL-P2 and Su-SL-P9 → Su-IL-P10 → Su-SL-P10. In both cases, the SL haplotypes are a subset of the IL haplotypes. Furthermore, unlike in the Co condition, we did not observe a dramatic contrasting pattern between the IL and SL samples. The haplotype frequencies are either declining or increasing relatively steadily throughout these two temporal sequences. The exceptions are (1) U30A/U82C, which disappeared in all the SL samples and appeared in all the IL samples (frequencies between 0.01 to 0.341), and (2) U30C/U34C and U30C/U34C/U82C, which only appeared in the IL-P10 sample with relatively high frequencies (0.059 and 0.021, respectively) (see Supplementary Table S3).

So far, comparisons of satBaMV haplotypes in the 5′ UTR revealed a possibility that mutations in this region could be beneficial to persistence in a local area but detrimental to long-distance dissemination. That is, genetic constraint, in the form of antagonistic pleiotropy, in the 5′ UTR (e.g., positions 30 and 82) could generate a trade-off between local replication and long-distance dissemination.

### Competition experiments

**Figure 7:**
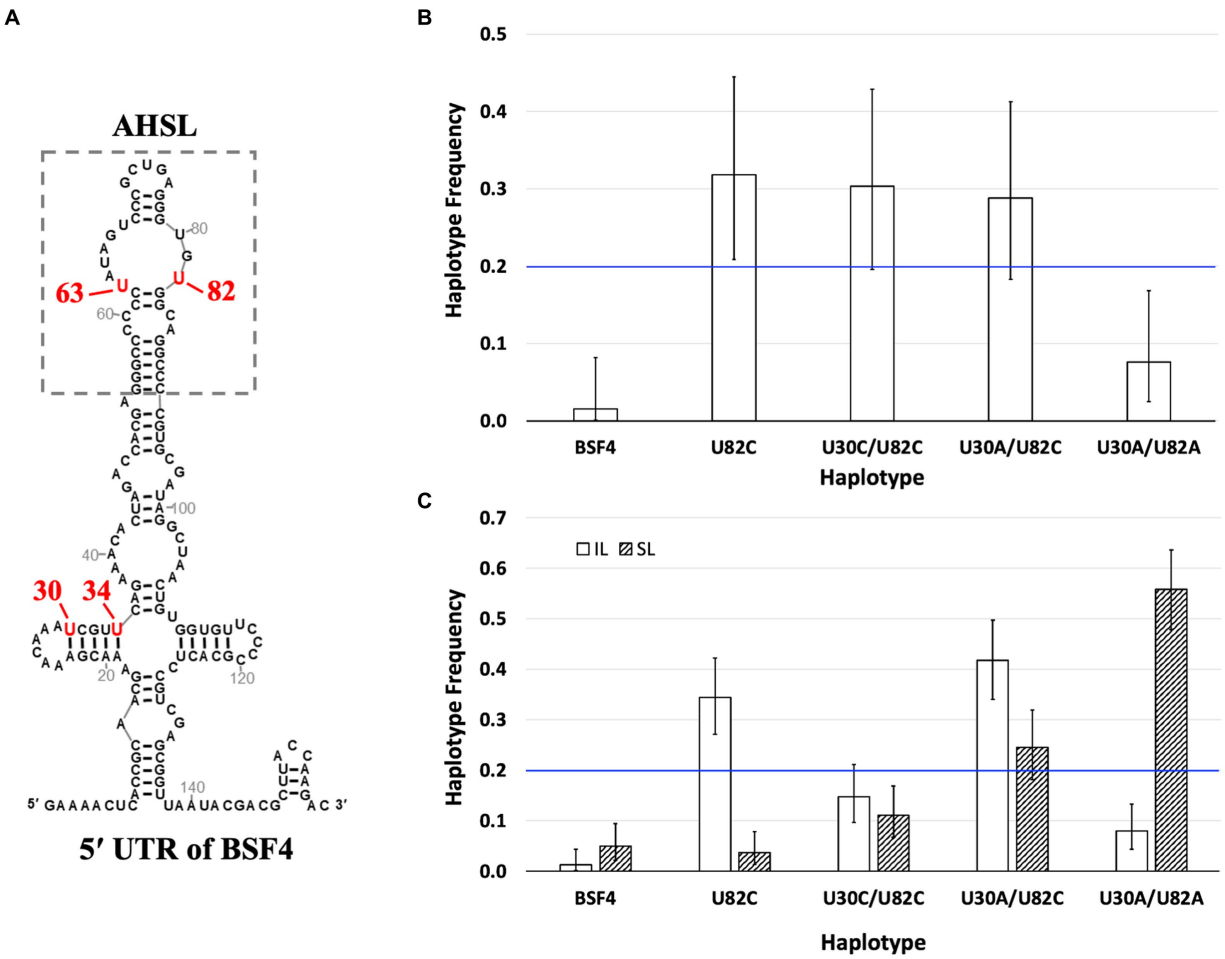
Competition of isogenic 5′ UTR haplotypes. **(A)** The secondary structures of 5′ UTR of satBaMV (Chen et al., 2012). The significant mutation sites after passaging and the substituted nucleotides are labeled in red. **(B)** Competition of satBaMV haplotypes in *N. benthamiana* protoplasts. An equal ratio of the listed five haplotypes was co-transfected with pBaORF1, which provides BaMV’s RdRp for genome replication. The satBaMV progenies were sampled 48 h after transfection. **(C)** Competition of satBaMV haplotypes in inoculation leaf (IL) and systemic leaf (SL). The listed five satBaMV haplotypes were co-inoculated with BaMV to *N. benthamiana* plants through agro-infiltration. Plant tissues were collected at 10 DPA for IL and 30 DPA for SL. For all competition experiments, the frequency of each satBaMV haplotype was enumerated by cloning and sequencing (see main text). Error bars indicate the 95% confidence intervals of the frequency estimates. The blue horizontal line at 0.2 shows the expected frequency when no competitive difference exists.

To determine whether mutations we observed in the 5′ UTR during SPEs (Figure 7A) exhibit antagonistic pleiotropy, thus resulting in the trade-off between local replication and long-distance dissemination, we conducted several competition experiments using isogenic satBaMV strains constructed via site-directed mutagenesis. Specifically, four haplotypes were used: U82C, U30A/U82C, U30A/U82A, and U30C/U82C. These haplotypes are thereafter designated as UC, AC, AA, and CC, respectively.

We used the UNAFold service^2^ to investigate whether the mutations may affect the 5′ UTR secondary structure. All four haplotypes showed similar structures and free energies as that of BSF4 (Supplementary Figure S3), indicating the introduced mutations do not appreciably change the RNA stability. This result is corroborated by empirical studies as well. When *N. benthamiana* protoplasts supplied with pBaORF1 (providing BaMV’s RdRp) were separately infected, we found that each haplotype can accumulate to similar levels of genome RNA (Supplementary Figure S4) and P20 protein (Supplementary Figure S5) as those of the ancestral BSF4. These results demonstrate that the engineered mutations did not fundamentally alter each haplotype’s replication or translation ability.

Together with the ancestral BSF4 (UU), these five satBaMV strains were tested for their replication competence, local replication, and long-distance dissemination in (1) *N. benthamiana* protoplasts (Chen et al., 2012) and (2) *in planta* inoculation leaves (IL) and systemic leaves (SL).

(1) Competition in *N. benthamiana* protoplasts. The competition experiment was conducted by transfecting replicase-expressing protoplasts (the same as the single infection experiments) and mixtures with an equal amount of plasmids that encode each of the five satBaMV strains (providing satBaMV RNA transcripts). To determine the competition outcome, we sampled the resulting satBaMV population by cloning and determined the identity of each individual via sequencing. The competition experiment was repeated three times. Although only a small number of clones (18–24) were sequenced, Fischer’s exact test of independence showed that the ratios of these haplotypes were not significantly different among the three replicates (*p* = 0.5437), thus allowing the pooling of the data for a better statistical analysis. An exact test of the goodness-of-fit of the pooled data showed that the ratio of haplotype frequency significantly deviates from the expected equal ratio (for a total of 66 clones, the observed UU:UC:CC:AC:AA = 1:21:20:19:5, *p* = 7.248 × 10^−7^), indicating differential replication competence among these haplotypes during mixed infection. *Post hoc* analysis using the exact binomial test showed that both the ancestral BSF4 [0.015 (0.000–0.082, 95% CI)] and U30A/U82A [0.076 (0.025–0.168)] have lower than expected frequencies after the 48-h incubation period while the haplotype U82C has a higher-than-expected frequency [0.318 (0.209–0.444)]. The other two haplotypes, U30C/U82C [0.303 (0.196–0.429)] and U30A/U82C [0.288 (0.183–0.413)] showed frequencies as expected (i.e., the 95% confidence intervals include the frequency of 0.200). Figure 7B shows the estimated haplotype frequencies and 95% confidence intervals. It appears that haplotypes with the U82C mutation are more competitive in replication than those without it.

(2) Competition *in planta*. To test whether these haplotypes differ in their ability to persist in the IL and long-distance dissemination to SL, we co-inoculated BaMV and an equal proportion of satBaMV mix onto *N. benthamiana* using agroinfiltration. Plant tissues were collected at the same intervals as those used during the SPEs, i.e., 10 DPI for IL and 30 DPI for SL. The frequency of each haplotype is again sampled with cloning and enumerated by sequencing. After Fisher’s exact test of independence, showing non-significance (IL: *p* = 0.4053; SL: *p* = 0.7382), the number of each haplotype from all three replicates was pooled together. An exact test of goodness-of-fit showed that both haplotype ratios significantly deviate from the expected equal proportion (result for IL: UU:UC:CC:AC:AA = 2:56:24:68:13, *p* = 6.785 × 10^−23^; for SL: UU:UC:CC:AC:AA = 8:6:18:40:91, *p* = 3.308 × 10^−29^). The exact binomial test was used to conduct *post hoc* analyses and determine the estimated proportion for each haplotype.

Among these haplotypes, the ancestral BSF4 showed a consistent decline in its frequency in both the IL and the SL [0.012 (0.002–0.044) and 0.0491 (0.021–0.094), respectively]. Two haplotypes showed opposite frequency trends in the IL and SL conditions: U82C has a higher frequency in IL [0.343 (0.271–0.422)] but lower in SL [0.037 (0.014–0.078)], while U30A/U82A has the opposite trend [IL: 0.080 (0.043–0.133); SL: 0.558 (0.479–0.636)]. The rest two haplotypes showed a similar pattern of remaining as expected in one condition while displaying an increased or decreased frequency in the other. For U30C/U82C, its frequency remained as expected in the IL condition [0.147 (0.097–0.211)] but declined in the SL condition [0.110 (0.067–0.169)]. For U30A/U82C, its frequency increased in the IL condition [0.417 (0.341–0.497)] but remained the same in the SL condition [0.245 (0.182–0.319)]. Figure 7C summarizes all the competition results.

These competition experiments showed that the ancestral BSF4 is at a competitive disadvantage in the presence of other mutations in positions 30 and 82. Its lower replication competence in protoplasts is directly translated to a lower presence in the IL (which requires some local dispersal to nearby cells) and SL. Some mutations exhibit a trade-off of home (IL) versus away (SL) advantages. For example, U82C mutation has a higher replication competence and is likely translated into its advantage in persisting in the IL. But such an advantage becomes a disadvantage when the systemic leaves are examined. The complete opposite is true for the U30A/U82A haplotype, which has a lower replication competence and thus is translated to a disadvantage in the IL. But, somehow, such a disadvantage becomes a great advantage for long-distance dissemination to the SL. This unexpected result suggests that the advantage of long-distance dissemination is not simply achieved by having more replications in the IL, followed by dissemination to the SL. A different mechanism(s) is needed to explain how different satBaMV strains compete to be disseminated to the entire plant.

## Discussion

### Infection ecology of a satellite RNA

To ensure an effective transmission from one plant host to another, we envision that a satRNA will need to fulfill a phenotypic demand—namely, maintaining as high a population frequency as possible throughout the entire infected plant (i.e., systemic infection). We reason that any genotype that can meet such a demand will have a higher chance of being transmitted to the next host plant. Consequently, the selective pressure, as a first approximation, is likely to be exerted on two aspects of a satRNA’s biology: genome replication and within-host dissemination; both activities depend on the resources (e.g., replicase and MP) provided by its cognate HV (Hu et al., 2009; Palukaitis, 2016). Therefore, the evolution of satRNA will be driven by competition for resources, either against other satRNA variants or against its HV and associated variants. However, the selective environment becomes more complicated when infection ecology is considered. For example, during the host-to-host spread, satRNA (together with its HV) may encounter an uninfected plant (coinfection, Co) or one that has already been infected (superinfection, Su). Regarding resource availability, these two scenarios could potentially pose different molecular environments for the invading satRNA. In the case of coinfection, the availability of each resource depends on the timing of its cognate HV’s developmental program. Presumably, the resource is scarce and somewhat delayed, at least initially, but there may be fewer competitors for the same resources. On the other hand, in the case of superinfection, the invading satRNA will find itself inside a cell that has already been occupied by resident HVs. The resources may be immediate and abundant, for there would be many HVs producing them, but it may also come with the price of many more competitors already *in situ*. Such is the caricatured “reality” a satRNA will face.

### Are coinfection and superinfection different selective environments?

It is not clear which infection scenario is more advantageous to an invading satRNA; however, our study showed that the accumulation of satBaMV is suppressed more under the superinfection condition (see Figure 2), suggesting that the presence of many more BaMV competitors at the initial infection sites is detrimental to satBaMV’s fitness. This observation implies that satBaMV will be subjected to a stronger selective pressure for genome replication when under the superinfection condition. Since both 5′ and 3′ UTRs are critical for satBaMV replication (Cheng and Tsai, 1999; Chiu et al., 2002; Annamalai et al., 2003; Chen et al., 2003, 2007, 2012; Huang et al., 2009, 2010; Lin and Lin, 2017), we expected that these two genomic regions would be the main targets of natural selection for improving replication efficiency. Our result from the serial passage experiments indeed revealed the presence of mutations with high frequencies at the 5′ UTR but, curiously, not at the 3′ UTR (see Supplementary Tables S2, S3). Interestingly, a field survey of satBaMV isolates also showed the lack of sequence diversity at the 3′ UTRs (Yeh et al., 2004). One possible reason for the conserved sequence is likely due to the structure and sequence requirements for satBaMV replication (Huang et al., 2009). We interpreted the emergence and spread of these mutations in the satBaMV populations as a response to the above-mentioned selective pressure for replication efficiency. However, most mutations at the 5′ UTR are shared between the Co and Su scenarios, suggesting that either satBaMV may have a limited set of mutations at the 5′ UTR that can be called upon for increasing replication efficiency or that the laboratory infection scenarios are artificial, thus does not represent two different selective environments assumed by us.

Nevertheless, the comparison between infection scenarios yielded an unexpected observation: the frequency of ancestral BSF4 haplotype declined faster and to a lower extent under the Co scenario (see Figure 5). This observation suggests that the ancestral BSF4 is subjected to a more stressful competition for replication resource under the Co condition. One possible explanation is that during the serial passage experiment under the Co scenario, satBaMV coevolves with its HV, BaMV, unlike in the Su scenario, in which the BaMV genome remains static during the passages. That is, under the Co condition, the ancestral BSF4 is likely to experience progressively more severe competition for replication resource from the evolving BaMVs, which are themselves a response to the evolving satBaMVs.

For BaMV, possible evolutionary responses can be mutations in the *cis*-acting element of the BaMV genome, e.g., the 5′ UTR, that allows the BaMV more efficient sequestration of the replicase, or in BaMV’s replicase gene, in which mutations may render the replicase more discriminatory against satBaMV’s 5′ UTR. Both evolutionary pathways can potentially result in a more stringent molecular environment for satBaMV replication. But surprisingly, genome-wide sequencing of the corresponding BaMV populations under the coinfection scenario revealed that the overall genomic compositions of the BaMV populations remained relatively constant. For example, for the four Co-IL populations, the proportions of the BaMV genome that are polymorphic progressed from 0.00% (P1), 0.05% (P2), 0.55% (P6), to 0.61% (P10), and for the three Co-SL populations, the proportions are 0.14% (P1), 0.75% (P9), to 0.50% (P10) (see Supplementary Table S5). Moreover, most SNPs have low frequencies and do not persist throughout the entire passage. The great majority of the SNPs found in various BaMV populations are ephemeral. The only exceptions are eight SNPs found in the Co-SL P9 and P10 populations, with frequencies close to fixation (see Supplementary Table S5). These near-fixation SNPs are found in the 5′ UTR (1), *ORF1* (6), and *ORF2* (1) genes. However, closer examination of these *ORF1* and *ORF2* SNPs showed that none results in amino acid substitution. Therefore, the replicase protein will be identical to that of the ancestral BaMV strain. The only remaining near-fixation SNP at position 40 is located at the base of the putative ASHL, an important feature that contributes to replication competence (Chen et al., 2012). Since only the Co-SL experiment has this mutation, therefore, it cannot be the reason why the frequency of the ancestral BSF4 declined faster in both Co-IL and Co-SL experiments. The faster decline of the BSF4 frequency may not be caused by coevolution between satBaMV and BaMV.

In summary, the seemingly convergent evolution of satBaMV’s 5′ UTR under both the coinfection and superinfection scenarios and the lack of BaMV evolution under the coinfection scenario suggest that the two seemingly different selective environments (i.e., coinfection vs. superinfection) are quite similar to each other, at least from the point-of-view of satBaMV. These results further imply that the dynamics of satBaMV’s haplotypes at the 5′ UTR may be mainly driven by competition within the satBaMV population rather than competition between satBaMV and BaMV populations.

### Antagonistic pleiotropy as the cause for the observed trade-off between local replication and long-distance dissemination

Antagonistic pleiotropy is a phenomenon in which a mutation is beneficial for the carrier in one life stage (or environment) but detrimental in another (Zhang and Wagner, 2013; Paaby and Rockman, 2013a,b). The conflicting evolutionary demands in different life stages (or environments) will inevitably result in a fitness trade-off. For viruses, trade-off due to antagonistic pleiotropy is not uncommon. However, most viral studies focus on trade-offs in adapting to different hosts, mainly manifested as antagonistic pleiotropy between viral attachment proteins and cell receptors (Duffy et al., 2006; Tumpey et al., 2007; Nikolin et al., 2012) or indirectly through changes in the regulatory sequences (Presloid et al., 2008). For plant viruses, antagonistic pleiotropy is also thought to be the main cause for various observed trade-offs when adapting to different host strains or species (Garcia-Arenal and Fraile, 2013).

In our current study, comparing satBaMV populations between IL and SL revealed a striking pattern of 5′ UTR haplotypes that dominated either IL or SL. We interpreted the pattern of contrasting differences as being consistent with the hypothesis that there is a trade-off between local replication and long-distance dissemination. That is, genotypes that are better at genome replication are worse at long-distance dissemination, and vice versa. We tested this hypothesis by competing five isogenic satBaMV strains (including the parental BSF4) that only differed in positions 30 and 82 (see Figure 7A), as revealed in the SPEs. Since all five isogenic strains were premixed together before inoculation on the same leaf, we can treat them as at the same starting line. Therefore, the distribution of each haplotype in different parts of the infected host plant should reflect two traits: replication competence and the tendency for long-distance dissemination. If there is no trade-off between these two traits, then the haplotype with the highest replication competence should also be the one with the highest frequency in the distal tissues of the plant. However, it is clear from Figure 7B that, when compared to the rest, the ancestral BSF4 (U30/U82) performs the worst across all conditions (protoplast, IL, and SL). Therefore, it is not surprising that during the SPEs, the ancestral haplotype is quickly overtaken by the U82C haplotype, which performs much better at IL (and, unsurprisingly, in the protoplasts as well) but worse at the SL. Contrastingly, the U30A/U82A haplotype has the opposite performance, with it being much better at the SL but much worse at the IL and protoplasts. Interestingly, for the intermediate haplotypes between these two opposites, U30A/U82C seems to perform better under all three conditions when compared to the alternate U30C/U82C (see Figures 7B,C).

What mechanism(s) could have contributed to the apparent trade-off between replication and movement? It has been established that the systemic movement of the BaMV RNA genome is in the form of a ribonucleoprotein (RNP) complex that comprises at least viral-encoded MPs (TGBp1, TGBp2, and TGBp3) and CP (Chou et al., 2013). We can reasonably assume that satBaMV also exploits a similar complex formation for its movement (Chang et al., 2016). Besides the BaMV-dependent movement, satBaMV also engages in BaMV-independent movement, involving the formation of a different RNP complex that comprises its P20 and host fibrillarin protein (Chang et al., 2016). In either case, it is likely that the satBaMV genome is fully associated with proteins, either from the viral (Lee et al., 2011; Chou et al., 2013) or the host (Huang et al., 2017a,b; Alazem et al., 2020) origins. On the other hand, for the replication-competence RNP complex, the presence of viral replicase is essential. We can hypothesize that satBaMV has at least two main forms of RNP complex, one for replication and the other for movement. The trade-off between replication and movement is thus mediated through the distribution of satBaMV RNAs in these two complexes. For the U82C haplotype, most RNAs would be engaged with the replicase; therefore, most of them are in the replication complex. For the U30A/U82A haplotype, the opposite is true. The U30A/U82A haplotype is thus preferentially trafficked to distal tissues of the plant, resulting in the observed pattern of more of the haplotype being sampled at the systemic leaves. Since replication and movement complexes are common, we believe this phenomenon could be universal among plant viruses.

### Are movement-advantageous haplotypes relevant in satBaMV evolution?

Although we have identified the presence of movement-advantageous genotypes, we believe that these genotypes may be short-lived. That is, given a long enough time, coupled with the high mutation rate characteristic of a quasispecies population, the mutations responsible for movement advantage will quickly be replaced by mutations, such as the U82C, that confer higher replication rates. Therefore, for a chronic infection like satBaMV, mutations that allow for better movement will eventually be replaced by mutations responsible for better replication, irrespective of whether the replication-advantageous mutations are trafficked from the initial infection site or eventually emerge *in situ*. Despite being evolutionarily transient, these movement-advantageous genotypes could nevertheless impact satBaMV evolution. During the initial stage of infection, the preferentially trafficked satBaMV genotypes are like early founders who bring with them not only the movement-advantageous mutations but also all other mutations in the genome that happened to hitchhike along. Such a genetic hitchhiking may contribute to the overall satBaMV sequence diversity even if the replication-advantageous mutations eventually outcompeted the original movement-advantageous mutations.

### Is there a replication-dissemination trade-off in the field?

Upon re-examination of our field samples (Wang et al., 2014), we found that there was no significant polymorphism detected at the four positions (30, 34, 63, and 82) within the 5′ UTR (Supplementary Table S6), except for the nearly ubiquitous presence of the U82C mutation. However, more than a couple of other mutations were present in samples from different bamboo species and locations (data not shown). This observation could be attributed to either the transient nature of mutations at positions 30/34/63/82, as hypothesized earlier, or it may be that the specific mutations identified during our serial passage experiments are primarily an adaptation to the *N. benthamiana* tobacco plant, which is significantly different from the natural bamboo hosts. Nevertheless, our proposed trade-off between local replication and long-distance dissemination may still operate in the bamboo hosts, albeit with a different set of antagonistic pleiotropy mutations. It will be of great interest to determine whether satBaMV also exhibits a similar trade-off in a monocot host, such as *Brachypodium distachyon* (Liou et al., 2014).

## Data availability statement

The datasets presented in this study can be found in online repositories. The names of the repository/repositories and accession number(s) can be found below: https://www.ncbi.nlm.nih.gov/, SAMN32554463-SAMN32554477, SAMN32609748-SAMN32609757.

## Author contributions

S-CL, M-RL, Y-HH, I-NW, and N-SL contributed to conception and design of this study. S-CL and M-RL performed the experiments. S-CL and I-NW organized and analyzed the amplicon datasets and results. I-NW performed the statistical analysis. S-CL, I-NW, and N-SL wrote the manuscript. All authors contributed to the article and approved the submitted version.

## Funding

This work was supported by Thematic Research Program from Academia Sinica, Taiwan (AS-103-TP-B01) to N-SL and I-NW and the Academia Sinica Investigator Award, Taiwan (AS-IA-108-05), to N-SL.

## Acknowledgments

The authors would like to thank Mei-Yeh Lu (High Throughput Genomics Core, Biodiversity Research Center, Academia Sinica, Taiwan) for performing Illumina sequencing, Wen-Dar Lin (Bioinformatics Core Lab, Institute of Plant and Microbial Biology, Academia Sinica, Taiwan) for bioinformatics analysis, Mei-Jane Fang (Genomic Technology Core Laboratory, Institute of Plant and Microbial Biology, Academia Sinica, Taiwan) for Sanger DNA sequencing analysis, and Jhih-Wei Wang and Meng-Hsun He for their technical supports.

## Conflict of interest

The authors declare that the research was conducted in the absence of any commercial or financial relationships that could be construed as a potential conflict of interest.

## Publisher’s note

All claims expressed in this article are solely those of the authors and do not necessarily represent those of their affiliated organizations, or those of the publisher, the editors and the reviewers. Any product that may be evaluated in this article, or claim that may be made by its manufacturer, is not guaranteed or endorsed by the publisher.

## Footnotes

^1^ http://rackj.sourceforge.net/

^2^ http://www.unafold.org/mfold/applications/rna-folding-form-v2.php
