# Supplementary material for: Trade-off between local replication and long-distance dissemination during experimental evolution of a satellite RNA
Source: Front Microbiol. 2023 Aug 4;14:1139447. doi: 10.3389/fmicb.2023.1139447 (PMC10436602; doi:10.3389/fmicb.2023.1139447)
Supplement: Supplementary file 2 [file Presentation_1.PPTX]

## Slide 1
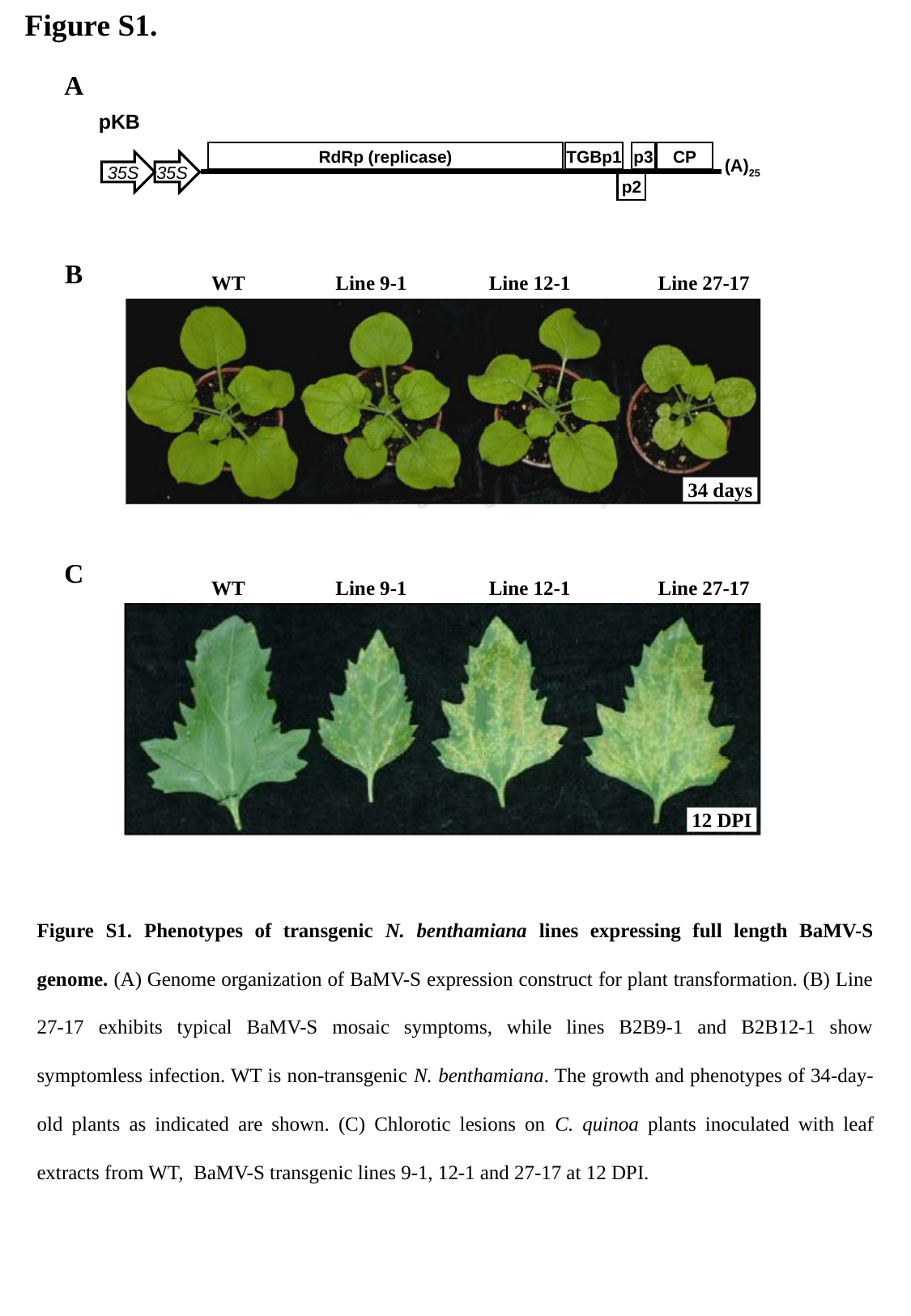

Figure S1.
A
pKB
RdRp (replicase)
TGBp1
p3
CP
p2
(A)25
35S
35S
B
WT
Line 9-1
Line 12-1
Line 27-17
34 days
C
WT
Line 9-1
Line 12-1
Line 27-17
12 DPI
Figure S1. Phenotypes of transgenic N. benthamiana lines expressing full length BaMV-S genome. (A) Genome organization of BaMV-S expression construct for plant transformation. (B) Line 27-17 exhibits typical BaMV-S mosaic symptoms, while lines B2B9-1 and B2B12-1 show symptomless infection. WT is non-transgenic N. benthamiana. The growth and phenotypes of 34-day-old plants as indicated are shown. (C) Chlorotic lesions on C. quinoa plants inoculated with leaf extracts from WT, BaMV-S transgenic lines 9-1, 12-1 and 27-17 at 12 DPI.

## Slide 2
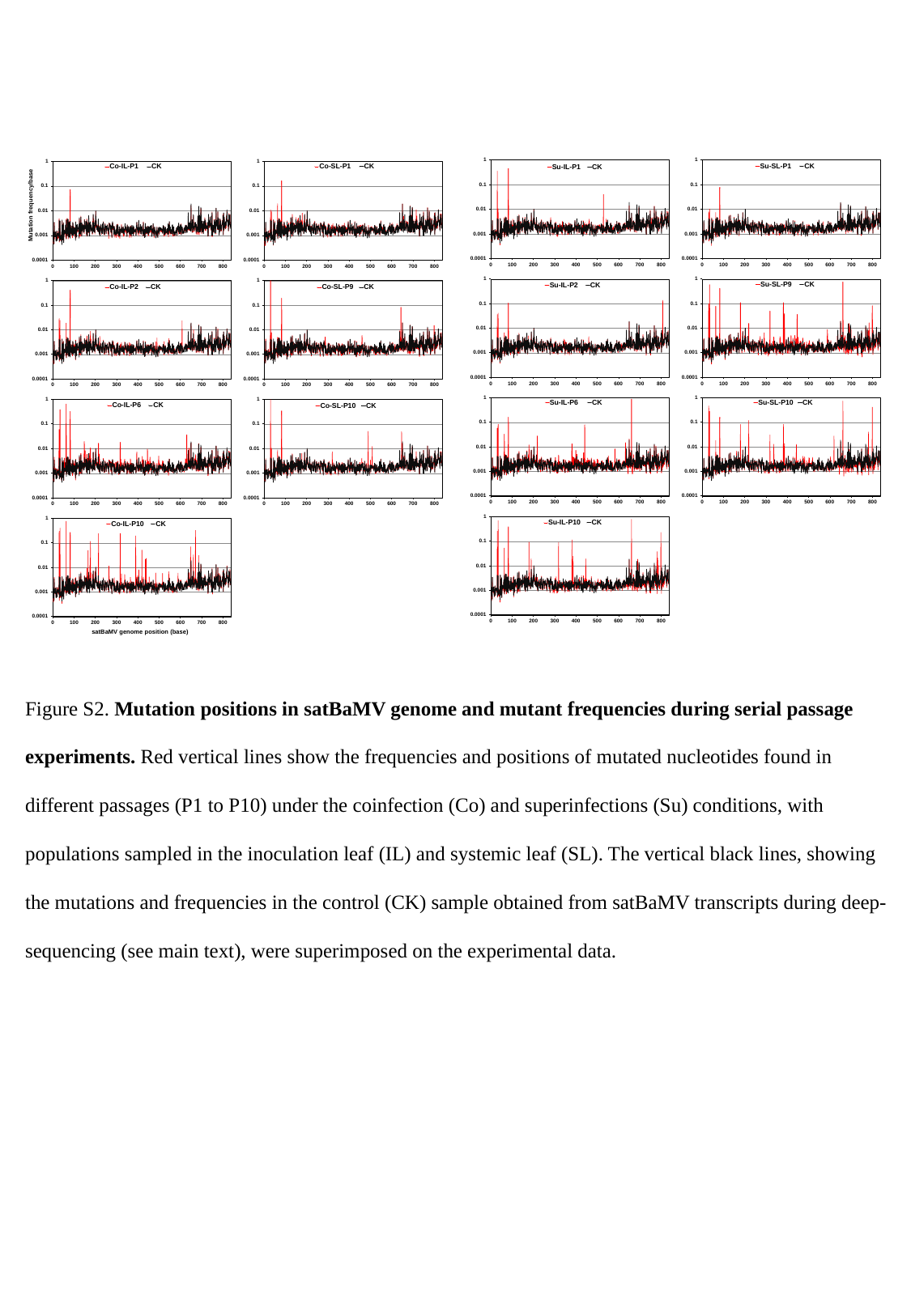

Figure S2. Mutation positions in satBaMV genome and mutant frequencies during serial passage experiments. Red vertical lines show the frequencies and positions of mutated nucleotides found in different passages (P1 to P10) under the coinfection (Co) and superinfections (Su) conditions, with populations sampled in the inoculation leaf (IL) and systemic leaf (SL). The vertical black lines, showing the mutations and frequencies in the control (CK) sample obtained from satBaMV transcripts during deep-sequencing (see main text), were superimposed on the experimental data.

## Slide 3
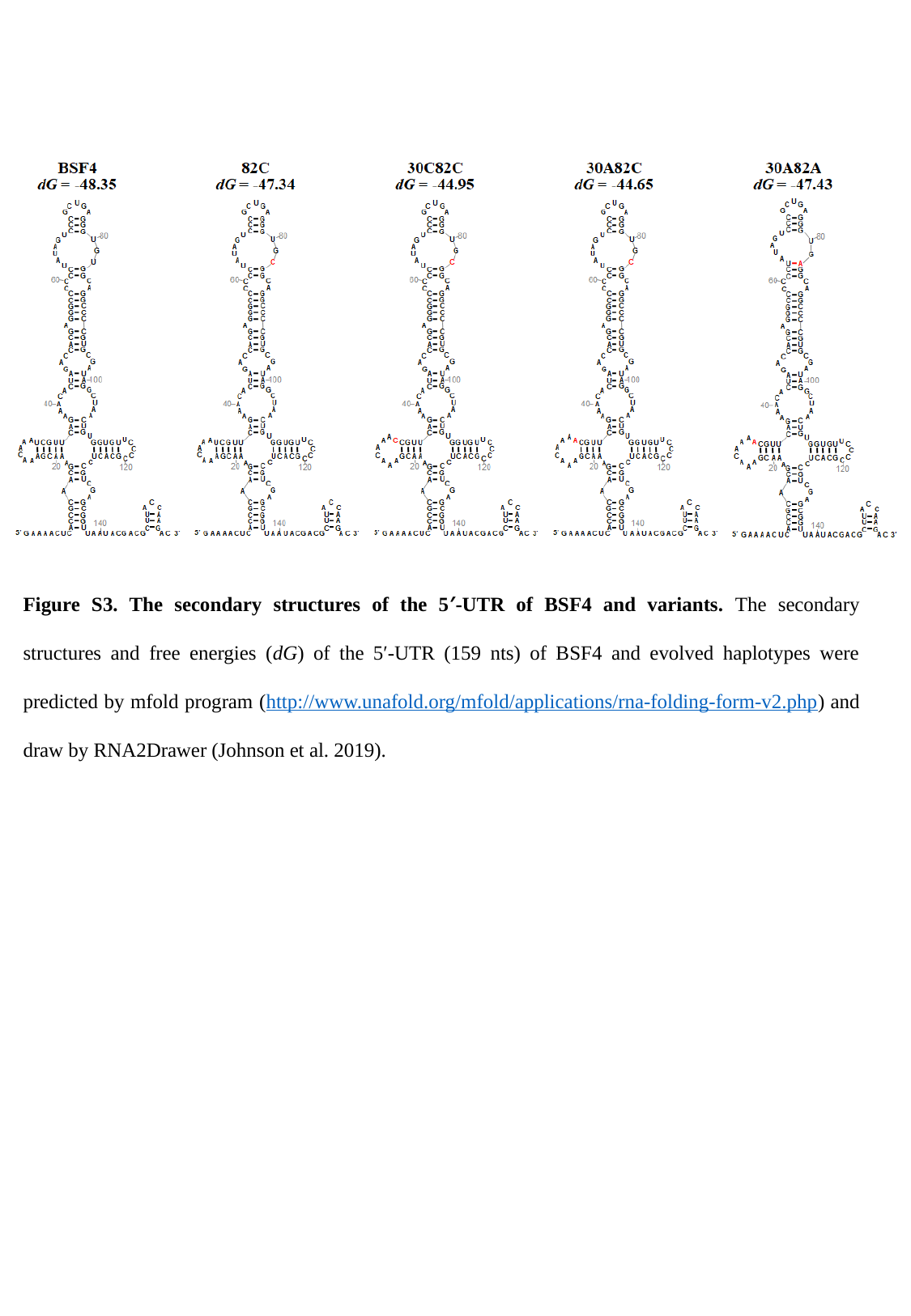

Figure S3. The secondary structures of the 5′-UTR of BSF4 and variants. The secondary structures and free energies (dG) of the 5′-UTR (159 nts) of BSF4 and evolved haplotypes were predicted by mfold program (http://www.unafold.org/mfold/applications/rna-folding-form-v2.php) and draw by RNA2Drawer (Johnson et al. 2019).

## Slide 4
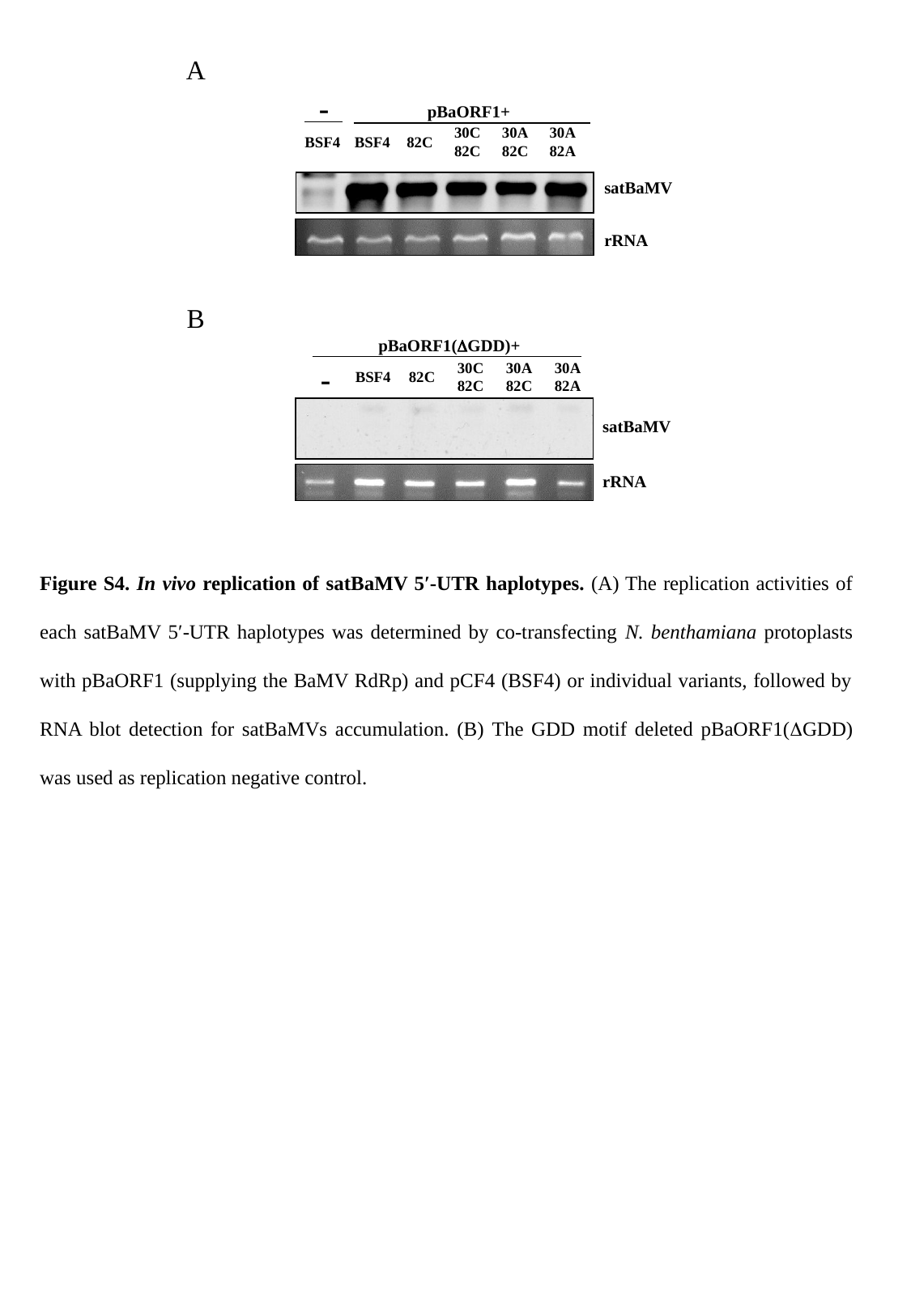

A
-
pBaORF1+
30C
82C
30A
82C
30A
82A
BSF4
BSF4
82C
satBaMV
rRNA
B
pBaORF1(DGDD)+
30C
82C
30A
82C
30A
82A
-
BSF4
82C
satBaMV
rRNA
Figure S4. In vivo replication of satBaMV 5′-UTR haplotypes. (A) The replication activities of each satBaMV 5′-UTR haplotypes was determined by co-transfecting N. benthamiana protoplasts with pBaORF1 (supplying the BaMV RdRp) and pCF4 (BSF4) or individual variants, followed by RNA blot detection for satBaMVs accumulation. (B) The GDD motif deleted pBaORF1(ΔGDD) was used as replication negative control.

## Slide 5
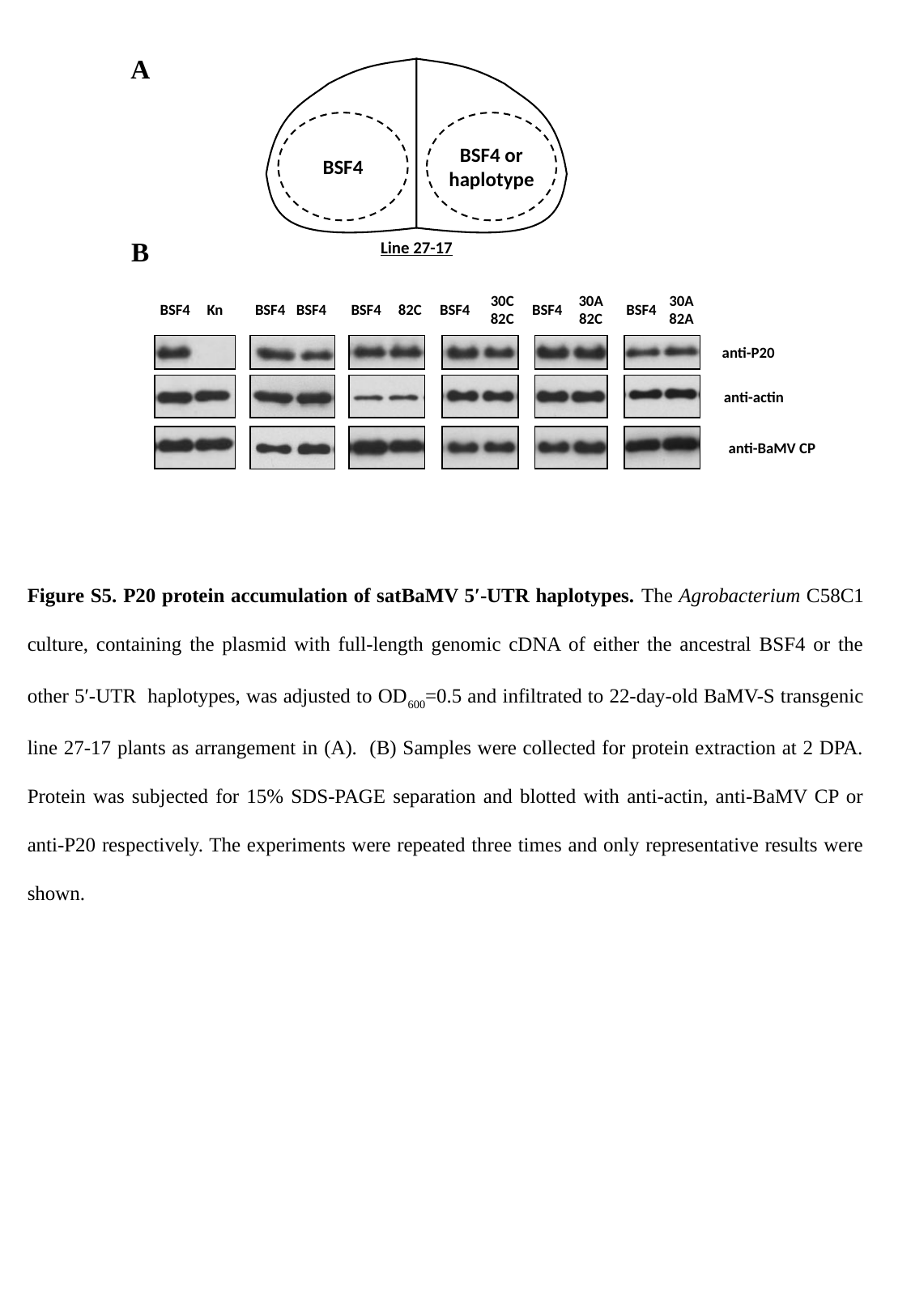

A
BSF4
BSF4 or
haplotype
Line 27-17
B
30C
82C
30A
82C
30A
82A
BSF4
Kn
BSF4
82C
BSF4
BSF4
BSF4
BSF4
BSF4
anti-P20
anti-actin
anti-BaMV CP
Figure S5. P20 protein accumulation of satBaMV 5′-UTR haplotypes. The Agrobacterium C58C1 culture, containing the plasmid with full-length genomic cDNA of either the ancestral BSF4 or the other 5′-UTR haplotypes, was adjusted to OD600=0.5 and infiltrated to 22-day-old BaMV-S transgenic line 27-17 plants as arrangement in (A). (B) Samples were collected for protein extraction at 2 DPA. Protein was subjected for 15% SDS-PAGE separation and blotted with anti-actin, anti-BaMV CP or anti-P20 respectively. The experiments were repeated three times and only representative results were shown.
